# Probiotic Bacteria and Their Cell Walls Induce Th1-Type Immunity Against *Salmonella* Typhimurium Challenge

**DOI:** 10.3389/fimmu.2021.660854

**Published:** 2021-05-12

**Authors:** José María Lemme-Dumit, Silvia Inés Cazorla, Gabriela Del Valle Perdigón, Carolina Maldonado-Galdeano

**Affiliations:** ^1^ Laboratorio de Inmunología, Centro de Referencia para Lactobacilos (CERELA-CONICET), San Miguel de Tucumán, Argentina; ^2^ Cátedra de Inmunología, Facultad de Bioquímica, Química y Farmacia, Universidad Nacional de Tucumán, San Miguel de Tucumán, Argentina

**Keywords:** probiotic, cell wall, immunomodulation, Th1-type response, *Salmonella* Typhimurium, mucosal adjuvant

## Abstract

Probiotics have been associated with a variety of health benefits. They can act as adjuvant to enhance specific immune response. Bacterial cell wall (CW) molecules are key structures that interact with host receptors promoting probiotic effects. The adjuvant capacity underlying this sub-cellular fraction purified from *Lactobacillus casei* CRL431 and *L. paracasei* CNCMI-1518 remains to be characterized. We interrogated the molecular and cellular events after oral feeding with probiotic-derived CW in addition to heat-inactivated *Salmonella* Typhimurium and their subsequent protective capacity against *S.* Typhimurium challenge. Intact probiotic bacteria were orally administered for comparison. We find that previous oral feeding with probiotics or their sub-cellular fraction reduce bacterial burden in spleen and liver after *Salmonella* challenge. Antibody responses after pathogen challenge were negligible, characterized by not major changes in the antibody-mediated phagocytic activity, and in the levels of total and *Salmonella*-specific intestinal sIgA and serum IgG, respectively. Conversely, the beneficial effect of probiotic-derived CW after *S.* Typhimurium challenge were ascribed to a Th1-type cell-mediated immunity which was characterized by augmentation of the delayed-type hypersensitivity response. The cell-mediated immunity associated with the oral feeding with probiotic-derived CW was accompanied with a Th1-cell polarizing cytokines, distinguished by increase IFN-γ/IL-4 ratio. Similar results were observed with the intact probiotics. Our study identified molecular events associated with the oral administration of sub-cellular structures derived from probiotics and their adjuvant capacity to exert immune modulatory function.

## Introduction

The intestinal microbiota is a complex microorganism ecosystem that plays essential roles in both digestive and the gut immune function (1, 2). As part of this community, beneficial microorganisms termed probiotics, are able to improve host health (3–5). Probiotics can influence the intestinal immune system by several mechanisms including change in the microbiota composition and its function, and improvement of the intestinal epithelial barrier (6, 7). Probiotics can activate the gut immune system by direct interaction with the intestinal epithelial and immune cells and modulate lamina propria and extraintestinal immune cell functions (8–10). In addition, one of the most extensively effects documented for certain probiotic strains are their capacity to exclude or inhibit enteric pathogens such as *Salmonella* (11–15).

*Salmonella* spp. remains one of the main agents causing foodborne disease in the worldwide (16). *Salmonella* infection is self-limited to gastrointestinal illness; although, in immunocompromised hosts, the enteric pathogen can translocate the intestinal barrier and spreads systemically causing severe disease (17–19). Mice are susceptible to *Salmonella* infection and have been ample used to investigate bacteria pathogenesis and host immune response (20, 21). Several reports have demonstrated that probiotic administration to mice before and after oral challenge with *S.* Typhimurium, improve animal survival, reduce pathogen spread outside the intestine, attenuate intestinal inflammation, and modulate cytokines production and IgA secretion in the gut (14, 15, 22). Furthermore, probiotic administration, has shown increases antimicrobial activity of the intestinal fluid against *S.* Typhimurium, which was associated with disruption of the enteric bacteria cell wall (CW) and its fragmentation (23).

The CW of Gram positive probiotic bacteria, exhibit a peptidoglycan layer embedded with teichoic acid motifs and polysaccharide molecules (24). Bacterial CW molecules are crucial structures involved in the probiotic effects (25). These structural ligands act as microbes-associated molecular patterns (MAMPs) that interact with host pattern-recognition receptors (PRRs) and activate immune and non-immune cells (26). We have previously shown that administration of probiotic-derived CW to mice for 7 days, modulate cytokine secretion of the intestinal epithelial cells, enhance antimicrobial function of macrophages at different compartments including Peyer’s patches, peritoneal cavity, and spleen, and increase the number of IgA-secreting cells in the gut lamina propria (10). Despite the beneficial effect of probiotics and their sub-cellular fraction on the innate immune function, remains not well characterized the contribution of the probiotic CW as oral adjuvant of the innate and adaptive immune response.

Due to the scarceness of effective and safety oral adjuvant available, our aim was to analyze the adjuvant effect of two probiotic strains *Lactobacillus casei* CRL 431 (Lc431) and *L. paracasei* CNCM I-1518 (Lp1518) and their CW (CW431 and CW1518, respectively) in an immunization process with heat-inactivated *S.* Typhimurium, mimicking a prophylactic vaccine protocol. We evaluate the protective capacity of the immune response elicited by the immunogen heat-inactivated *S.* Typhimurium adjuvanted with probiotics or their CW against *S.* Typhimurium challenge. The immune response was characterized by measuring antibody levels, cytokine production and cell-mediated function of mice at day 21 post-immunization and following *S.* Typhimurium challenge.

## Material and Methods

### Animals

Male BALB/c mice (6-weeks-old; weight, 26 ± 4 g) were obtained from closed random bred colony maintained at CERELA (Centro de Referencia para Lactobacilos, San Miguel de Tucumán, Tucumán, Argentina). Animals were kept during an interval period of 21 days in a controlled atmosphere (22 ± 2°C; 55 ± 2% relative humidity) with a 12h light/dark cycle, fed with a conventional balanced diet, and drinking water *ad libitum.* Studies were approved by CERELA Institutional Animal Care and Use Committee (Protocol No. CRL-BIOT-LI-2011/1A).

### Bacterial Strains and Probiotic CW

Probiotic strains: *Lactobacillus casei* CRL431 (Lc431) was obtained from CERELA culture collection, and *Lactobacillus paracasei* CNCM I-1518 (Lp1518) was provided by DANONE Argentina. These strains were cultured for 16h at 37°C in Man-Rogosa-Sharpe (MRS) broth (Britania, Buenos Aires, Argentina). *Salmonella enterica* serovar Typhimurium was obtained from Bacteriology Department, Hospital del Niño Jesús (San Miguel de Tucumán, Argentina) and cultured for 16h at 37°C in Brain Heart Infusion (BHI) broth (Britania, Buenos Aires, Argentina).

Probiotic CW were obtained as previously described (10). Briefly, Lc431 and Lp1518 were grown in MRS broth for 16h at 37°C. The biomass was harvested at 9,900 x*g* for 10 min at 4°C and washed three times with distilled sterile cold water. The cells were resuspended in one volume of distilled sterile cold water and harvested at 33,000 x*g* for 30 min. The pellet was lysed three times with a French press at 20,000 psi. The product obtained was harvested at 4,000 x*g* for 15 min at 4°C. The supernatant obtained was them harvested at 30,000 x*g* for 30 min at 4°C. The pellet was delipidated by sequent treatment with methanol:chloroform:water (1:1:1), methanol:chloroform (1:1) and chloroform. The delipidated product was resuspended in sterile 0.01M PBS pH 7.4 and treated with bovine pancreatic DNase I (50 µg/ml) and ribonuclease A (100 µg/ml) (Sigma-Aldrich, USA) by shaking at 37°C during 4h. The insoluble product (intact CW) was washed with distilled sterile water, and aliquoted of ~1000 µg/ml of total proteins determined by Bradford method. Intact CW was lyophilized (dry weight ~0.0080 g) and stored at -80°C until used. Lyophilized was resuspended in distilled sterile water and administered at 8 μg/μl/mice, as previously reported (27). The products obtained were used as CW from *L. casei* CRL431 (CW431), and *L. paracasei* CNCM I-1518 (CW1518).

### *Salmonella* Typhimurium Inactivation

*S.* Typhimurium was grown at 37°C in BHI broth until reach a final concentration of ~1 x 10^9^ colony forming units (CFU)/ml. Bacteria harvested at 3,000 x*g* for 10 min were washed with sterile and cold 0.01M PBS pH 7.4. The bacterial pellet was suspended in sterile PBS and inactivated by three heating cycle of 1h at 60°C. Bacterial deaths was confirmed by plating on *Salmonella-Shigella* (SS) agar (Britania, Buenos Aires, Argentina). Protein concentration of heat-inactivated *S.* Typhimurium was determined by Bradford method, and aliquots of 100 µg/ml was stored at -20°C until used.

### Feeding Procedures

Lc431 and Lp1518 suspensions were administrated for 7 and 5 consecutive days, respectively, optimal time for the activation of the intestinal immune system for each strain, as previously described (28). Probiotic bacteria were given in 5 ml of sterile 10% (w/v) skim milk powder and resuspended in the drinking water to a final concentration of 1x10^8^ CFU/ml. One hundred microliters/day/mice of CW431 or CW1518 (8 μg/μl) were given by *gavage* during 7 consecutive days. Animals receiving PBS served as control.

### Study Design

**Figure 1:**
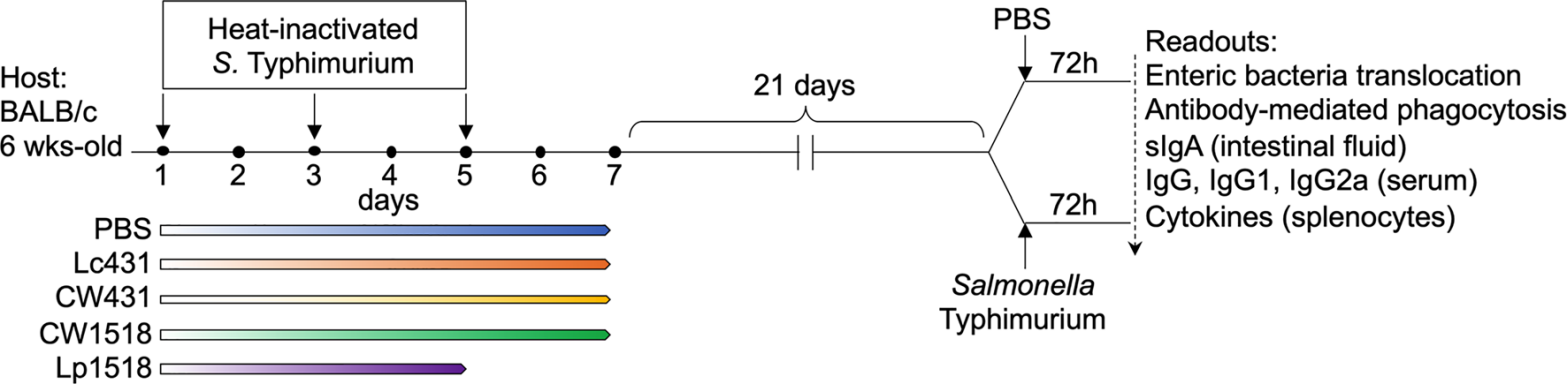
Study design. BALB/c mice (6 weeks-old) were given probiotic bacteria, Lc431 or Lp1518, in the drinking water for 7 and 5 consecutive days, respectively. CW431 or CW1518, were administered by gavage for 7 consecutive days. Mice were immunized with heat-inactivated *S.* Typhimurium *via* oral route on days 1, 3, and 5 of either probiotics or CW administration. After 21 days, animals were challenged with one dose of virulent *S.* Typhimurium *via* oral route and a group of mice received PBS (oral) and served as control (uninfected); mice were euthanized 72h.

**Figure 1** shown the experimental design. The study contained five experimental groups, with three to six mouse per group, were treated with: PBS, or Lc431, or CW431, or Lp1518, or CW1518 as described above. Mice were immunized with heat-inactivated *S.* Typhimurium (100 μl) *via* oral route on day 1, 3, and 5 of the aforementioned treatment. After 21 days, a group of mice were orally challenged with virulent *S.* Typhimurium (1x10^9^CFU/ml) whereas other group of animals received PBS (uninfected). All animals were euthanized 72h post-challenge.

### Enteric Bacteria Colonization in Spleen and Liver

The spleen and liver were aseptically removed, weighed, and homogenized under sterile conditions using a microhomogenizer (MSE, England) in 5 ml of peptone water (0.1%). Serial dilutions were plated on MRS and MacConkey agar (Britania, Buenos Aires, Argentina) and incubated overnight at 37°C.

### Opsonophagocytic Uptake by Macrophages

Opsonophagocytic assay was performed as it was described (29), with minor modifications. An overnight culture of *S.* Typhimurium was diluted 1:25 in fresh BHI broth and incubated at 37°C until the OD_600_ reached 0.6. Ninety microliters of this bacterial suspension were then added to 10 µl mice sera previously heat-inactivated at 56°C for 20 min, and incubated at room temperature (RT) for 20 min. Bacteria suspension (1x10^7^ CFU) was added to the semi-confluent macrophage cell line, Raw 264.7 in RPMI-1640 plus 10% FBS, to a ratio 100:1 (bacterium/macrophage). The plate was incubated for 45 min (37°C, 5% CO_2_). Macrophages were washed with sterile PBS, replaced with fresh RPMI-1640 medium containing 100 µg/ml gentamicin and incubated additionally for 1h (37°C, 5% CO_2_). After removing the media, macrophages were washed three times with sterile PBS. Finally, the Raw 264.7 cells were lysed with 500 µl of 0.5% Triton X-100 and vigorous pipetting, and cell lysates were plated on SS agar. Intracellular bacteria were enumerated. The percentage of bacteria taken up by Raw 264.7 cells (i.e., percent opsonization) was calculated by dividing the number of bacteria that survived the gentamicin treatment by the inoculum size and multiplying by 100.

### Total and *Salmonella*-Specific Antibody Measurements

Serum (IgG, IgG1, and IgG2a) and intestinal fluid (sIgA) anti-*S.* Typhimurium antibodies were quantified by ELISA. Briefly, 96-wells EIA/RIA plates high binding (Costar, USA) were coated with 100 µl/well of heat-killed *S.* Typhimurium suspension (10^9^ CFU/ml) in carbonate-bicarbonate buffer pH 9.6 and incubated overnight at 4°C. Non-specific protein-binding sites were blocked with PBS containing 5% skim milk powder for 1h at 37°C. Appropriate sample dilutions (serum, 1:1000; intestinal fluid, 1:25) were incubated for 1h at 37°C. For IgG and subclasses detection, primary antibodies included biotinylated mouse anti-IgG (Jackson ImmunoResearch, USA; 1:20,000), anti-IgG1 and -IgG2a (BD Pharmingen, USA; 1:250) were incubated for 1h at RT. Secondary antibodies included HRP-labelled antibodies obtained in rabbit specific for mouse IgG, IgG1, and IgG2a were incubated for 1h at 37°C. For sIgA detection, peroxidase-conjugated mouse anti-sIgA (Sigma-Aldrich, USA) were incubated for 1h at 37°C. Antibodies were detected by 3,3’,5,5’-tetramethylbenzidine (TMB) peroxidase substrate (BD Biosciences, USA). Plates were incubated for 30 min at RT and reaction was stopped with 2N H_2_SO_4_. Plates were read at 450 nm using a VERSA Max Microplate Reader (Molecular Devices, Sunnyvale, CA, USA). Total sIgA was determined in the intestinal fluid as previously described (30). Briefly, affinity-purified monoclonal goat anti-IgA (α-chain specific Sigma, St Louis, MO, USA) was added in 0.05 M carbonate-bicarbonate buffer (pH 9.6) to 96 wells plates and incubated at 37°C for 1h. The plates were then washed with PBS containing 0.05% Tween 20 (PBS-T) and blocked for 1h at 37°C with 0.5% non-fat dry milk in PBS. Plates were washed and incubated for 2 h at 37°C with either 50 μl of standard kappa IgA (Sigma, St. Louis, USA) or 50 μl of intestinal fluid samples. Plates were washed and incubated in the presence of HRP-conjugated anti-IgA-specific antibodies (Sigma, St. Louis, USA) for 1h at 37°C. Plates were washed and TMB was added. Plates were incubated for 30 min at RT; reaction were stopped with 2N H_2_SO_4_ and plates were read at 450 nm.

### Delayed-Type Hypersensitivity (DTH) Assay

Mice were intradermally injected with 10 µg of heat-inactivated *S.* Typhimurium in one of the footpads. As control, the contralateral footpad was injected with an equal volume of saline solution. A group of mice were orally challenged with virulent *S.* Typhimurium (1x10^9^CFU/ml) whereas other group of animals received PBS 24h after intradermal injection. Footpad swelling was measured 48h post injection using a digital caliper with a precision of 0.01 mm. The increase in footpad thickness (mm) was calculated as the footpad thickness injected with heat-killed *S.* Typhimurium – the footpad thickness injected with saline solution.

### Cytokine Measurements

The spleen was aseptically removed and transferred into 15 ml tubes containing 5 ml of Hank’s buffered salt solution (HBSS) (Sigma-Aldrich, USA) and 10% fetal bovine serum (FBS). The organ was mechanically dissociated under sterile conditions. Cells were harvested by centrifugation at 800-1000 x*g* for 15 min at 4°C. The cell pellets were gently mixed with 2 ml of sterile red blood lysing buffer for 2 min. The hemolysis was stopped with sterile PBS. Cells were centrifuged and resuspended in RPMI-1640 medium (Gibco/Life Technologies, USA) containing 10% FBS. Total splenocytes were cultured at a concentration of 1x10^6^ cells/ml in 24-well plates with fresh RPMI-1640 medium containing 10% FBS and gentamicin (100 µg/ml). The plates were incubated for 16h (37°C, 5% CO_2_). IFN-γ, TNF-α, IL-4, IL-6, and IL-10 were measured in the supernatant of splenocytes by ELISA following the manufacturer’s instructions (BD OptEIA; BD Biosciences, USA).

### Statistical Analysis

Statistical significances were calculated using non-parametric Mann-Whitney *U* test to compare two groups. Significant differences among more than two groups were determined based on one-way ANOVA with Šidák’s correction for multiple comparison. *P* values, and number of samples are indicated in the figure legends. Statistical tests were performed and plotted using GraphPad Prism v9.

## Results

### Probiotic Bacteria and Their CW Reduce Enteric Bacteria Burden in Spleen and Liver After *S.* Typhimurium Infection

We first evaluated whether oral administration of probiotics or their CW along with oral immunization with heat-inactivated *S.* Typhimurium generate an immune response able to protect mice against *S.* Typhimurium challenge. Enteric bacteria spread was determined in extraintestinal organs including liver and spleen. As expected, uninfected animals were cleared of organisms in spleen and liver. By contrast, in *Salmonella*-challenged mice, enteric organisms were enumerated in the macerated of spleen and liver (**Table 1**). Interestingly, infected animals that have received Lc431 or Lp1518 or their CW, enteric organisms load were significantly reduced in spleen and liver compared to PBS group (**Table 1**). These results suggest that probiotic bacteria and their CW protect the host, reducing enteric bacteria burden after *S.* Typhimurium challenge.

**Table 1:** Enteric bacteria burden into spleen and liver after S. Typhimurium challenge.

Organ	Group	MRS	MacConkey
*Spleen*	PBS	6.831 ± 0.056	6.742 ± 0.028
	Lc431	6.283 ± 0.155*	5.983 ± 0.172*
	CW431	6.165 ± 0.154*	5.669 ± 0.269**
	Lp1518	6.197 ± 0.152*	5.719 ± 0.077**
	CW1518	6.294 ± 0.030*	6.077 ± 0.150*
*Liver*	PBS	6.677 ± 0.127	6.591 ± 0.164
	Lc431	5.853 ± 0.096*	5.460 ± 0.087*
	CW431	5.926 ± 0.074*	5.692 ± 0.107^ns^
	Lp1518	5.998 ± 0.246*	5.493 ± 0.466*
	CW1518	5.925 ± 0.173*	5.444 ± 0.306*

CFU are expressed as log_10_ numbers of bacteria per gram of organ. Each value represents the mean ± SEM (n=6). One-way ANOVA with Šidak correction for multiple comparisons. *P < 0.05; **P < 0.01; ns, not significant compared to PBS group.

### Probiotics and Their CW Do Not Improve Antibody-Mediated Opsonophagocytosis Against *S.* Typhimurium

**Figure 2:**
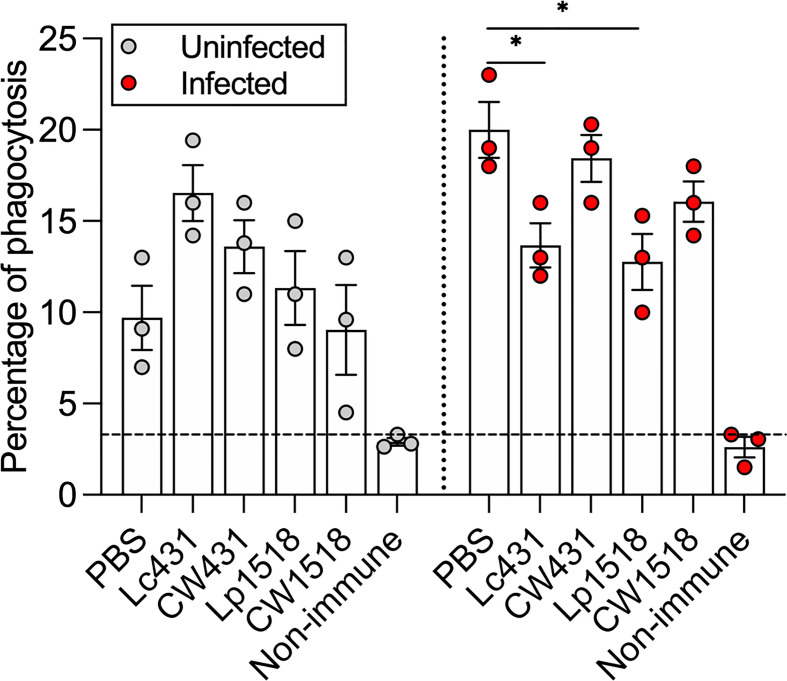
Probiotic and their CW did not enhance antibody-mediated phagocytosis against *S.* Typhimurium. Percentage of phagocytosis of *S.* Typhimurium opsonized with serum obtained from uninfected or infected animals. Non-immunized animals (littermate mice) were used as cut-off (dashed line). Data are shown as mean ± SEM from three independent experiments with 3 mice per group. *P* values were calculated with one-way ANOVA with Šidak correction for multiple comparisons. *P < 0.05.

We evaluated whether antibodies produced during oral administration of Lc431 or Lp1518 or their CW along with oral immunization with heat-inactivated *S.* Typhimurium improve the capacity of phagocytic cells to uptake *S.* Typhimurium. Mice serum were collected on day 21 after administration and pre-incubated with S. Typhimurium. Opsonized *Salmonella* were then incubated with phagocytic cells, Raw264.7. Serum derived from PBS group and non-immunized mice, were used as control. Increased opsonophagocytic activity was observed in serum derived from both uninfected and *Salmonella*-infected mice compared to non-immunized mice (**Figure 2**). Striking, in *Salmonella*-infected mice that received probiotic bacteria Lc431 or Lp1518, decreased phagocytosis was observed compared to PBS group (**Figure 2**). Thus, probiotics and their CW did not enhance antibody-mediated phagocytosis against *S.* Typhimurium.

### Probiotics and Their CW Modulate Antibody Immune Response Elicited Against *S.* Typhimurium Challenge

**Figure 3:**
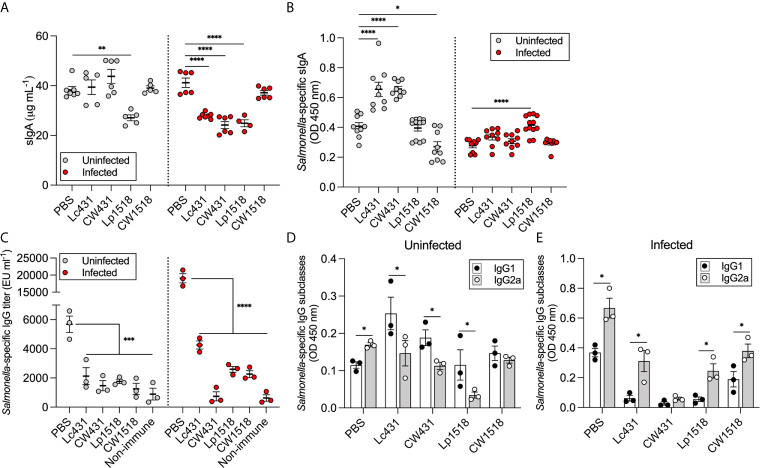
Probiotic and their CW modulate mucosal antibody response against *S.* Typhimurium. **(A, B)** Total and *Salmonella*-specific sIgA levels in intestinal fluid of uninfected and *S.* Typhimurium infected mice. **(C)** *Salmonella*-specific IgG titer in serum of uninfected and *S.* Typhimurium infected mice. **(D, E)** *Salmonella*-specific IgG subclasses determined in serum of uninfected **(D)** and *S.* Typhimurium infected **(E)** mice. Data are shown as mean ± SEM. Data are pooled from two independent experiments **(A, B)** or representative of three independent experiments **(C–E)** with 3-6 mice per group. *P* values were calculated with one-way ANOVA with Šidak correction for multiple comparisons **(A–C)** and non-parametric Mann-Whitney test **(D, E)**. *P < 0.05; **P < 0.01; ***P < 0.001; ****P < 0.0001.

We next investigated the levels of antibodies elicited after *S.* Typhimurium challenge in mice that were given probiotics or their CW along with oral immunization with heat-inactivated *S.* Typhimurium. Secretory IgA (sIgA) and IgG levels were determined by ELISA in the intestinal fluid and serum, respectively. sIgA levels were similar with all but one treatment (Lp1518) in uninfected mice compared to PBS group; whereas, during infection reduced levels of sIgA were detected in Lc431-, CW431-, and Lp1518-treated mice as compared to PBS (**Figure 3A**). Moreover, *Salmonella*-specific sIgA levels were increased in uninfected mice that received Lc431 and CW431, while reduced levels were determined in CW1518 in comparison to PBS group (**Figure 3B**). After *Salmonella* challenge, a significantly increase of *Salmonella*-specific sIgA were detected in Lp1518-treated mice as compared to PBS as well as, increased levels (although not significant) where observed for Lc431-, and CW431-treated mice compared to PBS group (**Figure 3B**). A striking observation was the reduced titers of *Salmonella*-specific IgG in mice that received probiotics or their CW, even after *Salmonella* challenge compared to PBS group (**Figure 3C**).

Further, we determined the levels of IgG1 and IgG2a in the serum of mice as a bias of Th2/Th1 response, respectively (31). Increased levels of IgG1 were detected in uninfected mice that received probiotics or their CW as compared to IgG2a levels (**Figure 3D**); inversely, after *Salmonella* challenge, increased levels of IgG2a were detected in mice that received Lc431 or Lp1518 or their CW (**Figure 3E**).

### Probiotics and Their CW Improve *In Vivo* Cellular Immune Response

**Figure 4:**
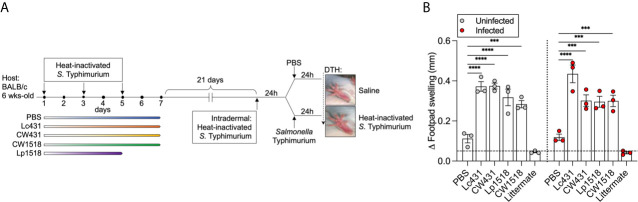
Probiotic and their CW enhance Th1 response against *S.* Typhimurium. **(A)** Intradermal injection with heat-inactivated *S.* Typhimurium were performed in one footpad at day 21 of probiotic or CW administration and oral immunization with heat-inactivated S. Typhimurium as was described in **Figure 1**. PBS was injected in the contralateral footpad as control. After 24h, animals were challenged with one dose of virulent *S.* Typhimurium *via* oral route and a group of mice received PBS (oral) and served as control (uninfected); mice were euthanized 48h from intradermal injection. **(B)** Footpad swelling measurement of uninfected and *S.* Typhimurium infected mice. Non-immunized animals (littermate mice) were used as cut-off (dashed line). Data are shown as mean ± SEM from three independent experiments with 3 mice per group. *P* values were calculated with one-way ANOVA with Šidak correction for multiple comparisons. ***P < 0.001; ****P < 0.0001.

We next sought to investigate whether Lc431 or Lp1518 or their CW promote cell-mediated response against *S.* Typhimurium. We performed an *in vivo* DTH test as measurement of Th1 response. Intradermal injection of heat-inactivated *S.* Typhimurium was performed in one footpad of mice that have previously received oral administration of Lc431 or Lp1518 or their CW along with oral immunization of heat-inactivated *S.* Typhimurium. Following 24h, a group of mice were challenged with *S.* Typhimurium. Footpad swelling was measured after 48h of the intradermal injection (**Figure 4A**). We found increased swelling of the footpad in both uninfected and infected mice previous immunized with heat-inactivated *S.* Typhimurium and that received Lc431 or Lp1518 or their CW compared to both PBS and littermate mice (**Figure 4B**).

### Probiotics and Their CW Modulate Cytokine Production in a Strain Specific Manner

**Figure 5:**
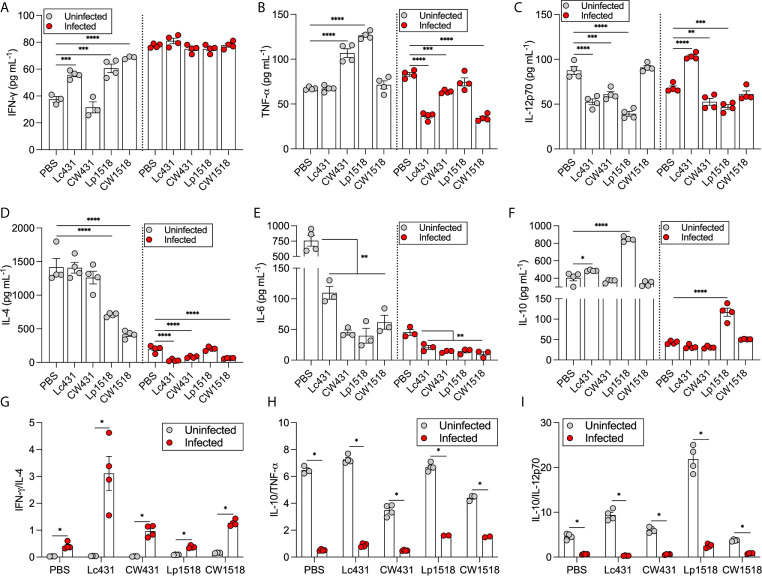
Probiotic and their CW modulate cytokine production. **(A–F)** Cytokines measured in the supernatant of splenocytes of uninfected and *S.* Typhimurium infected. Data are shown as mean ± SEM from three independent experiments with 3-4 mice per group. P values were calculated with one-way ANOVA with Šidak correction for multiple comparisons. *P < 0.05; **P < 0.01; ***P < 0.001; ****P < 0.0001. Cytokine ratios were calculated dividing IFN-γ levels by IL-4 **(G)**, and IL-10 by TNF-α **(H)** and IL-12p70 **(I)**. *P* values were calculated by Student’s *t* test. *P < 0.05.

Next, we evaluated the cytokine profiles in mice 21 days after treatments and orally challenged with *S.* Typhimurium. The levels of IFN-γ, TNF-α, IL-12p70, IL-4, IL-10, and IL-6 were measured in the supernatants of splenocytes by ELISA. Splenocytes of uninfected mice under all treatments but one (CW431) revealed increased levels of IFN-γ compared to PBS-treated animals; whereas *Salmonella*-infected mice, similar levels of IFN-γ were detected in the supernatants for all evaluated conditions as compared to PBS (**Figure 5A**). TNF-α levels were significantly increased in uninfected mice that received CW431 and Lp1518 treatment compared to PBS, while after *Salmonella* challenge, reduced levels of TNF-α were detected in Lc431-, CW431-, and CW1518-treated mice compared to PBS group (**Figure 5B**). IL-12p70 were reduced in the supernatant of splenocytes of uninfected mice that received Lc431, CW431, and Lp1518 as compared to PBS-treated mice; by contrast, *Salmonella*-challenged mice, IL-12p70 were increased in Lc431-, but reduced in CW431-, and Lp1518-treated mice compared to PBS (**Figure 5C**). IL-4 levels were reduced in uninfected mice that received Lp1518 and CW1518, whereas after *Salmonella* challenge, reduced levels were detected in all groups but one (Lp1518) as compared to PBS (**Figure 5D**). The levels of IL-6 were reduced in both uninfected and *Salmonella*-challenged mice for all treatments compared to PBS group (**Figure 5E**). IL-10 levels were increased in uninfected mice that received Lc431 and Lp1518 compared to PBS group; whereas in *Salmonella*-challenged mice, IL-10 levels were increased in Lp1518-treated mice compared to PBS (**Figure 5F**).

We determined IFN-γ/IL-4, and IL-10/TNF-α and IL-10/IL-12p70 ratios as a balance of Th1/Th2 immune response. As expected, *Salmonella*-challenged mice revealed bias toward Th1-cytokine profile associated with both probiotics and their CW administration (**Figure 5G**). Conversely, uninfected mice revealed a Th2-cytokine profile with an anti-inflammatory state where probiotic bacteria demonstrate highest ratios IL-10/TNF-α and IL-10/IL-12p70 compared to their CW (**Figures 5H, I**). Thus, probiotics and their CW administration were able to modulate cytokine production in a strain specific manner contributing bias the response toward a Th1 profile during *Salmonella* challenge.

### Immunomodulatory Role of Probiotics and Their CW Against *S.* Typhimurium

**Figure 6:**
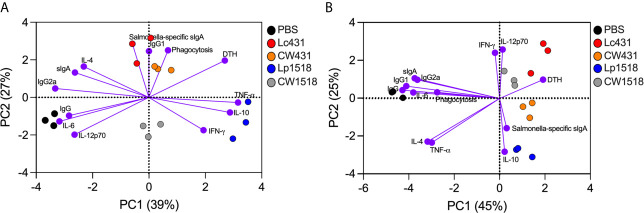
Probiotic and their CW reveal similar modulatory effect on the immune system during *S.* Typhimurium infection. Principal component analysis (PCA) plots of uninfected **(A)** and *S.* Typhimurium infected **(B)** mice. Variables analyzed: phagocytosis, sIgA, Salmonella-specific sIgA, IgG, IgG1, IgG2a, footpad swelling, IFN-γ, TNF-α, IL-12p70, IL-6, and IL-10.

To highlight the protective mechanisms driven by probiotics and their CW after *S.* Typhimurium challenge, we performed a principal component analysis (PCA) of the biological functions evaluated. We observed that Lc431- and CW431-treated mice clustered together and distantly from Lp1518, CW1518, and PBS group by principal component (PC) 1 on a PCA plot in uninfected mice (**Figure 6A**). Lc431/CW431 cluster was associated with *Salmonella*-specific sIgA, IgG1, and phagocytic activity function (**Figure 6A**). Lp1518 cluster was associated mainly to cytokine production such as IFN-γ, IL-10, and TNF-α; whereas CW1518 cluster was more closely to IL-12p70 and IFN-γ (**Figure 6A**). In addition, Lc431/CW431 and Lp1518 clusters were both related with cell-mediated response (as determined by DTH) (**Figure 6A**). Conversely, *Salmonella*-challenged mice, probiotics and their CW cluster together and distantly from PBS control by PC1 on a PCA plot (**Figure 6B**). As shown in **Figure 6B**, probiotics or their CW administration were associated with cell-mediated immunity, cytokine production (IFN-γ, IL-12p70, IL-10), and mucosal specific sIgA antibody.

## Discussion

Probiotic bacteria are beneficial microorganisms that contribute food digestion, modulate the intestinal microbial communities, suppress growth of pathogens, and enhance host immunity. Due to the scarceness of effective and safety oral adjuvant available, our aim was to analyze the adjuvant effect of probiotics and their CW in an immunization process with heat-inactivated *Salmonella*, mimicking a prophylactic vaccine protocol. We evaluate the protective capacity of the immune response elicited by the immunogen (heat-killed *S.* Typhimurium adjuvanted with probiotics or their CW) against a challenge with virulent *S.* Typhimurium. We selected two probiotic strains, Lc431 and Lp1518 based on their immunomodulatory properties. Lc431 has demonstrated adherence to and stimulate cytokine secretion of intestinal epithelial cells (7, 32), and both Lc431 and Lp1518 have shown to enhance phagocytic cell function, modulate pro-inflammatory cytokine production and increase IgA-expressing cells in the lamina propria of the small intestine (33), improve microbiota composition (28, 34), ameliorate allergic inflammation (35), and maintain immunity under stress conditions (36). In this work, we characterized the immune response elicited by the administration of probiotic-derived CW along with oral immunization with heat-inactivated *S.* Typhimurium. A side-by-side comparison with intact probiotic bacteria revealed similar effects.

*S.* Typhimurium is one of the major food poisoning agents causing mild gastroenteritis and diarrhea disease that self-limited to the gastrointestinal tract and affect humans at different ages around the world (37). Severe disease can be a life-threatening for young children, elderly, and immunocompromised host. A particular global concern is the emerge of multidrug-resistant by different *Salmonella* serotypes that difficult the treatment of individuals affected with severe disease (38, 39). Several studies have documented the protective effect of probiotics preventing *Salmonella* infections (13–15, 22, 40). *S.* Typhimurium infection was associated with disruption of tight junction and increased epithelial barrier permeability (41, 42). We found that oral administration of probiotic-derived CW reduced enteric bacteria translocation, in a similar manner as was accounted for the intact probiotic bacteria. It was reported that binding of MAMPs of Gram-positive bacteria to toll-like receptor 2 (TLR2) on the intestinal epithelial cells, suppress mucosal inflammation and maintain tight junction complex (43). In addition, purified S layer protein A (SlpA) from *L. acidophilus* NCK2187, regulate tight junction protein expression and prevent colitis *via* SIGNR3 signaling (44). Exposure of HT-29 and Caco-2 cell monolayers to *L. acidophilus* ATCC4356 and *Streptococcus thermophilus* ATCC19258, increased the expression of tight junction proteins occludin and ZO-1 on epithelial cells, that was correlated with decrease intestinal permeability (45). It remains to be elucidated whether probiotic-derived CW macromolecules from Lc431 and Lp1518 correlate with tight junction protein expression and maintain intestinal barrier function.

Probiotics exert several mechanisms of protection to the host including modulation of the mucosal and systemic immune responses. One of them encompass the capacity to impact humoral immune response. Antibodies are important players in host defense against pathogens by recognizing and binding to microorganisms or infected cells. In addition, probiotics have demonstrated to act as mucosal adjuvant by increasing immunogenicity to vaccines associated with higher magnitude in the antibody titers and protection (46, 47). Beyond their immediate role, binding and neutralizing antigens, antibodies can interact with cells of the innate immune system, leading to a remarkable diversity of effector functions to control and eradicate infections (48). For example, complement-fixing bactericidal antibodies can kill invasive *Salmonella*, and antibody-mediated opsonophagocytosis induce neutrophils oxidative burst which represent an additional arm of protection against the invasive enteric organism (49). In the current study, we found that oral administration of probiotics or their CW along with oral immunization with heat-inactivated *S.* Typhimurium slightly enhance antibody-mediated phagocytic function of macrophages against *S.* Typhimurium challenge.

At the mucosal tissues, IgA is the most abundant antibody isotype, although IgM and IgG are also present, the latest dominant in circulation (50). Oral immunization with an attenuated human rotavirus vaccine to neonatal gnotobiotic pigs co-colonized with the probiotic bacteria *L. rhamnosus* GG and *Bifidobacterium animalis lactis* Bb12, have shown enhanced mucosal IgA response and protection upon rotavirus challenge (51). Increased levels of total sIgA in the intestinal fluid of animals fed for 7 days with Lc431 and challenged with *S.* Typhimurium, were reported 10 days after pathogen infection (22). Similarly, Esvaran et al. (52) reported increased levels of *Salmonella*-specific intestinal IgA and serum IgG at day 10 post-challenge in animals that were given heat-killed *S.* Typhimurium *via* oral route and fed with *L. fermentum* PC2 for 5 days. In the current study, our data indicated that oral administration of probiotic-derived CW or the intact probiotic bacteria as adjuvant of heat-inactivated *S.* Typhimurium displayed a negligible mucosal IgA and serum IgG antibody response after 21 days and post-challenge with *Salmonella.* We speculate that oral administration of CW or their intact probiotic along with heat-inactivate *S.* Typhimurium generate polyreactive, low affinity IgA that could be below the limits of detection of the enzyme-linked immunosorbent assay (ELISA). Several studies have shown that non-invasive *Salmonella* was unable to induce intestinal IgA (53, 54). While our results do not contradict previous findings, where probiotics and their CW increased the number of IgA^+^ cells in the lamina propria after 7 days of administration (10), previous work have documented an average half-life for intestinal IgA plasma cells of 5 days and a maximum of 6 to 8 weeks (55). The development of an antigen-specific sIgA response is a long process that takes >14 days and around 3-4 weeks to detect an appreciable amount of sIgA in stool (56). Unlike IgG memory response, intestinal memory IgA response display an additive effect after repeated challenges, thus, a specific IgA response is limited to the persistent of a particular stimulus at any given time (57). This mechanism may contribute to maintain tolerance to commensal microorganisms. Together, this suggests that CW and probiotic may stimulate mucosal antibody response *via* T-independent mechanism that release transient low affinity intestinal IgA.

Martinoli and colleagues have shown that invasive and non-invasive *S.* Typhimurium can reach the spleen and elicited systemic IgG response (53). By contrast, our results showed a limited serum IgG response against *Salmonella* even when enteric bacteria reached liver and spleen. It has been reported significantly lower levels of rotavirus-specific IgG and total IgG in serum other than IgA in animals given probiotic bacteria, which suggest an immunomodulatory differential effect exerted by probiotic (51). We are currently studying the effects of probiotic-derived CW over time by determine kinetic of IgA- and IgG-secreting cells in the gut lamina propria, intestinal and serum IgA and IgG levels.

Despite the small antibody response generated by the oral immunization with heat-inactivated *S.* Typhimurium in addition to probiotics or their CW administration, we found that IgG2a was predominant after pathogen challenge. Importantly, we observed increased DTH response in animals that were given probiotic bacteria and their CW. Th1-type cell-mediated immunity promotes phagocytic cell activation and antimicrobial training. A handful of studies have demonstrated the ability of different probiotic strains to modulate Th1-type immune response (35, 58–61). Consistently, it was reported that Lc431 increased phagocytic function of macrophages in *S.* Typhimurium-infected mice (22).

An important observation was the Th1-cell polarizing cytokines in *S.* Typhimurium-challenged mice associated with the oral administration of probiotic-derived CW or the intact probiotic bacteria in addition to heat-inactivated *S.* Typhimurium. Th1 type-differentiated cells produce IFN-γ, which is essential during the initial phase of bacterial infection, and promote phagocyte-depend protective response, and suppress Th2-type humoral response (62, 63). Our findings showed that probiotic-derived CW and the whole probiotic bacteria exhibit similar levels of IFN-γ after *S.* Typhimurium challenge. This suggests that probiotics may maximize the Th1-type cell-mediated response preventing replication and spreading of the enteric pathogen. Consistently, Lc431 administration after *S.* Typhimurium infection, demonstrated elevate the frequencies of IFN-γ- and IL-10-secreting cells in the lamina propria and the presence of these cytokines in the intestinal fluid (14). In addition, it was reported elevated numbers of NK cells and IFN-γ levels in subjects consuming yogurt containing a mixture of probiotic bacteria (64). Several studies indicate that NK cells are predominant source of IFN-γ associated with protection against *S.* Typhimurium infection (65–67). By contrast, restraint of NK cell-produced IFN-γ by *Bacillus subtilis*-derived exopolysaccharide, reduces bacterial burden and inflammation during *Staphylococcus aureus* bloodstream infection (68). This suggests that probiotic modulate cytokine response in a strain- and pathogen-specific manner promoting host immunosurveillance. Furthermore, consistent with the Th1-biased response modulated by the administration of probiotic-derived CW and their intact bacteria against *S.* Typhimurium challenge, we found that IL-4 production was reduced. This is in line with the antibody outcomes observed.

Administration of probiotic-derived CW and the whole bacteria were able to modulate the secretion of other pro-inflammatory cytokines including TNF-α, IL-12p70, and IL-6 in the context of *S.* Typhimurium infection. Probiotics can suppress inflammation by modulating various signaling pathways including NF-κB and MAPK pathways involved in the production of pro-inflammatory cytokines (69). Primed antigen presenting cells with *L. jensenii* TL2937, modulate the production of pro- and anti-inflammatory cytokines in response to ETEC or LPS associated with negative regulators of TLR signaling (70). Moreover, probiotic-derived CW and the intact bacteria were able to modulate the secretion of IL-10, which demonstrated prevent damage inflammation caused by microbial pathogen infections (71). Further studies are needed to define the role of probiotic-derived CW interaction with pattern recognition receptors and signaling events.

Overall, our findings propose an immunomodulatory role for the sub-cellular fraction derived from probiotic bacteria after *S.* Typhimurium challenge. Their effects were comparable to the intact probiotic bacteria which may indicate their use as mucosal adjuvant. Importantly, probiotics-derived CW contribute to the acquired immune response against *S.* Typhimurium through the Th1-type cell-mediated immune response. Although, it is important to remark that each probiotic strain and its CW may evoke different outcomes. Given the immunomodulatory effects exerted for the probiotic-derived CW, this study opens new avenues to discern the exact component(s) on the CW of the adjuvant effect, although we do not exclude the possibility of the additive effect of different bacteria structures.

## Data Availability Statement

The raw data supporting the conclusions of this article will be made available by the authors, without undue reservation.

## Ethics Statement

Studies were approved by CERELA Institutional Animal Care and Use Committee. Written informed consent was obtained from the owners for the participation of their animals in this study.

## Author Contributions

JML-D, SIC, and CM-G designed, performed, analyzed and interpreted all the experiments. GP conceptualized the study, interpreted the data, and secured funding. All authors contributed to the article and approved the submitted version.

## Funding

This work was supported by grants from Consejo Nacional de Investigaciones Científicas y Técnicas (CONICET) Argentina (PIP No. 806), and Secretaría de Ciencia, Arte e Innovación Tecnológica (SCAIT), Universidad Nacional de Tucumán, Argentina (CIUNT 2964).

## Conflict of Interest

The authors declare that the research was conducted in the absence of any commercial or financial relationships that could be construed as a potential conflict of interest.
